# Advances in Waveguide Bragg Grating Structures, Platforms, and Applications: An Up-to-Date Appraisal

**DOI:** 10.3390/bios12070497

**Published:** 2022-07-08

**Authors:** Muhammad A. Butt, Nikolay L. Kazanskiy, Svetlana N. Khonina

**Affiliations:** 1 Institute of Microelectronics and Optoelectronics, Warsaw University of Technology, Koszykowa 75, 00-662 Warszawa, Poland; 2 Samara National Research University, 443086 Samara, Russia; kazanskiy@ipsiras.ru (N.L.K.); khonina@ipsiras.ru (S.N.K.); 3 IPSI RAS-Branch of the FSRC “Crystallography and Photonics” RAS, 443001 Samara, Russia

**Keywords:** Bragg grating, filter, sensor, plasmonics, metal-insulator-metal waveguide, silicon-on-insulator platform, polymer

## Abstract

A Bragg grating (BG) is a one-dimensional optical device that may reflect a specific wavelength of light while transmitting all others. It is created by the periodic fluctuation of the refractive index in the waveguide (WG). The reflectivity of a BG is specified by the index modulation profile. A Bragg grating is a flexible optical filter that has found broad use in several scientific and industrial domains due to its straightforward construction and distinctive filtering capacity. WG BGs are also widely utilized in sensing applications due to their easy integration and high sensitivity. Sensors that utilize optical signals for sensing have several benefits over conventional sensors that use electric signals to achieve detection, including being lighter, having a strong ability to resist electromagnetic interference, consuming less power, operating over a wider frequency range, performing consistently, operating at a high speed, and experiencing less loss and crosstalk. WG BGs are simple to include in chips and are compatible with complementary metal-oxide-semiconductor (CMOS) manufacturing processes. In this review, WG BG structures based on three major optical platforms including semiconductors, polymers, and plasmonics are discussed for filtering and sensing applications. Based on the desired application and available fabrication facilities, the optical platform is selected, which mainly regulates the device performance and footprint.

## 1. Introduction

Optical waveguides (WGs), in the traditional sense, are translucent geometries with a refractive index difference that directs optical beams via total internal reflection [1]. The most well-known illustration is optical fiber, which transmits light signals throughout the world with only a few tenths of a decibel per kilometer loss. Integrated photonic systems, in which optical WGs connect lasers, detectors, power splitters, filters, and modulators, are now the subject of significant study. From communications and information processing to monitoring and medicinal applications, these devices offer a wide range of uses [2]. Planar lightwave circuits are essential for the advancement of photonics current tech. They are two-dimensional photonic devices with numerous optical functionalities on a single chip. Photolithography is a technology for fabricating planar lightwave circuits that has evolved through time in the microelectronics sector [3]. In a cleanroom setting, ultraviolet (UV) radiation, selective etching (chemical or plasma), and doping (ion exchange or diffusion) are all part of the process. Photolithography, despite its benefits in reproducibility and parallel processing, is a time-consuming, stiff multistep operation that can often only generate 2D-optical WG circuits.

A Bragg grating (BG) structure is a regular WG with periodic refractive index (RI) variations running across it [4,5]. These aberrations create a one-dimensional photonic bandgap that reflects only a restricted spectrum of any broadband signal traveling through the WG. BG WGs are conceptually comparable to the well-known fiber Bragg gratings (FBGs) [6]. FBGs, which function as narrowband mirrors integrated into optical fibers and are widespread for wavelength division multiplexing (WDM), tunable filtering, and—when chirped—dispersion compensation in optical communications systems, have been made with lasers for nearly 30 years. Furthermore, because their resonant (reflected) wavelength is very sensitive to external factors such as temperature and strain, these devices are commonly utilized in sensing applications [4,7,8,9]. Ken Hill discovered FBG in 1978 at the Communication Research Centre in Canada [6,10]. Because of its excellent characteristics, such as its low cost, compact size, real-time response, high precision, high sensitivity, and electromagnetic interference, grating structures have gotten a lot of interest in the field of optical sensing since their invention. Using grating-based devices, it is possible to sense a variety of characteristics such as temperature, pressure, tension, and RI. High-temperature sensors, health and biological devices, structural engineering, industries, biochemical applications, radioactive environments, aerospace, marine, and civil engineering, and many more disciplines use FBGs today [11]. Because most glass materials have an *n_eff_* close to 1.5, Bragg response in the telecom band at 1550 nm requires a short grating period of about ~500 nm [12]. By holographic or phase mask interference, or by point-by-point writing, UV and ultrafast lasers may easily build precisely patterned BGs in optical fibers.

The introduction of arbitrarily shaped optical WGs, many of which are also arbitrarily inhomogeneous, dissipative, anisotropic, and/or nonlinear, has been a significant development in guided-wave optics, including fiber optics and integrated optics. To successfully design, optimize, and realize optical WGs, computational tools for modeling and simulation are crucial. Most of these WG arbitrariness instances do not lend themselves to analytical solutions. Numerous numerical approaches have been developed for this aim, such as the finite difference time domain (FDTD), finite element method (FEM), transfer matrix method (TMM), and coupled-mode theory (CMT). Particularly for the most comprehensive optical WG issue, the finite element method (FEM) is a strong and effective tool. Numerous optical waveguide issues would not be able to be solved without it given how extensively it is used in both industry and research. FEM has been used in the design and analysis of several optical components [12,13,14]. 

**Figure 1:**
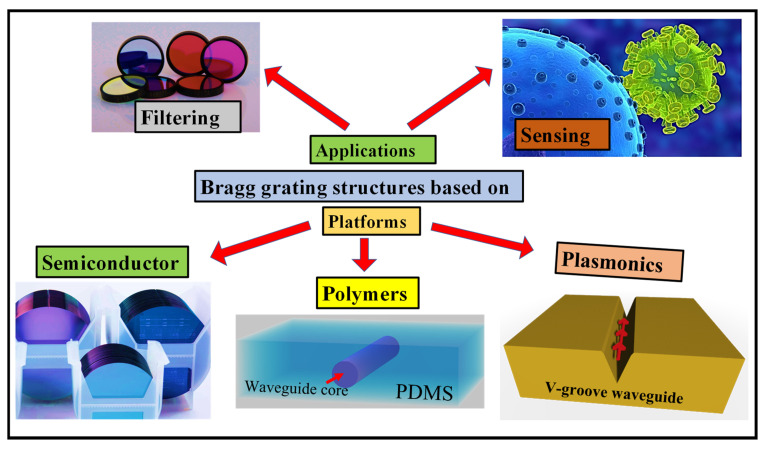
Outline of the review paper. BG structures are based on different platforms such as semiconductors [15], polymers, and plasmonics utilized in filtering [16] and sensing applications [17].

The paper is organized in the following manner: Section 2 provides an overview of the Bragg equation and provides the conditions to design a BG based on any suitable platform. In Section 3, recent advances in semiconductor BG structures are discussed for filtering and sensing applications. Silicon nitride (SiN) and silicon-on-insulator (SOI) are the most common semiconductor materials that are widely used for the realization of such vital components. Polymeric materials are widely used in optical applications, spanning from photovoltaics and light-emitting diodes (LEDs) to optical sensors, bioimaging with dye-labeled polymers, nonlinear optics, light-harvesting, and other optical subcomponents for structural color. In Section 4, novel polymer materials and the different fabrication methods for polymer WGs are discussed. Moreover, the recent advances in the polymer WG BG device are discussed for filtering and sensing applications. Although photonics present an appealing resolution to electronics’ speed limitations, one of the primary challenges in implementing photonic integrated circuits is shrinking the size of heavy photonic components. Plasmonic circuits, which closely restrict electromagnetic waves at the metal–dielectric contact, might offer a solution. In Section 5, the fundamentals of plasmonics, the types of plasmonic WGs, vital plasmonic materials, and BG devices based on the plasmonic platform for filtering and sensing applications are discussed. The paper ends with a brief conclusion stated in Section 6. The outline of the review paper is demonstrated in Figure 1.

## 2. Bragg Concept

**Figure 2:**
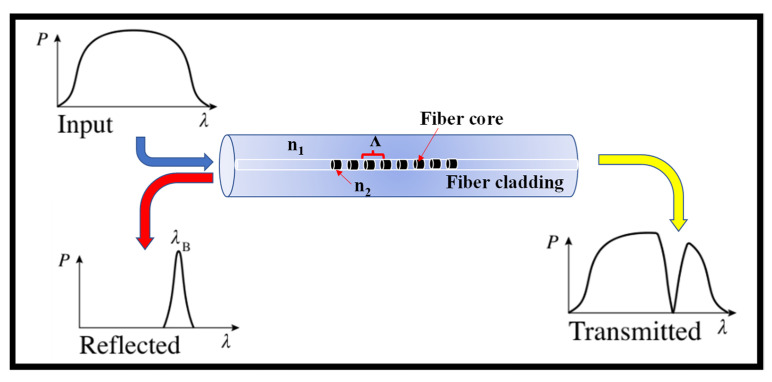
FBG structure and its spectral response.

When a light wave passes through a grating medium, it encounters minor Fresnel reflections at the interface of each RI perturbation. When all the individual reflections are in phase, constructive interference is created, and the medium reflects the incident wave throughout a strictly regulated wavelength range. The resonant wavelength for reflection where the mode coupling is maximum is provided by the classic Bragg equation in the basic scenario of coupling between two contrary propagating guided modes by a binary grating [18]:

$$\lambda_{Bragg}=\frac{2\times n_{eff}\;\Lambda }{m}\;;$$

where *λ_Bragg_* is the Bragg wavelength, *n_eff_* is the propagating mode’s effective modal index, Λ is the RI modulation’s period, and *m* = 1, 2, 3 is the diffraction, resonance, or grating order. Only non-sinusoidal RI variations result in higher-order WG BG resonances, and only odd higher-order modes are reflected if the grating has a 50 percent duty cycle square-wave RI form [19]. This is owing to the character of the periodic index function’s Fourier series expansion. The working mechanism of the WG BG structure is similar to the FBGs; therefore, a standard FBG and its spectral response are shown in Figure 2.

A fiber grating’s reflection bandwidth, which is normally far below 1 nm, is influenced by the length and intensity of the refractive index modulation. Long gratings with weak index modulations yield the smallest bandwidth values, which are suitable to produce single-frequency fiber lasers or for some optical filters, for example. Large bandwidths may be attained using both prolonged aperiodic designs and short, powerful gratings. BGs can be employed in temperature and strain sensors since the wavelength of maximum reflectance varies with temperature and mechanical strain in addition to the period of the BG. Transverse stress causes birefringence and, consequently, polarization-dependent Bragg wavelengths—for instance, when a fiber grating is squeezed between two flat plates. The high sensitivity, multiplexing capability, compact size, light weight, multi-modal sensing capability, immunity to electromagnetic interference, and low fabrication cost are only a few of the benefits of optical fiber grating-based sensors [20]. LPG, EFBG, tilted FBG, microstructured FBG, Photonic Crystal Fibers (PCF), LPG inscribed in PCF, and tilted FBG paired with SPR are a few examples of optical fiber grating-based biosensors that are known to operate according to various operating principles [21,22]. Due to their label-free refractive index measurement capabilities, optical grating sensors such as LPG, EFBG, and tilted FBG sensors are becoming more and more important in the development of chemo- and biosensors. To create thrombin biosensors, some of these FBG biosensor designs have been studied [23,24,25].

## 3. BG Structures Based on a Semiconductor Platform

In this section, BG structures based on the Si platform are discussed. This platform is interesting due to its high optical, physical, and chemical properties. The high refractive index contrast enables the formation of devices with a small footprint. 

### 3.1. Fundamentals of Si-Based BG Structures

BGs have developed into key optical devices and have been widely employed in numerous systems since the discovery of Bragg’s law in 1913 [26]. The desire to investigate on-chip integrated gratings derives from the swift expansion of Si photonics, which is enabling revolutionary applications through small circuits made using CMOS-compatible manufacturing technologies [27,28,29,30,31]. Gratings made of Si [32,33], SiN [34], and InGaAs/AlInAs [35] have been proven in a range of shapes, comprising variations and pillars on the strip and ridge formations, with the necessity to adjust attributes for each application driving the variety of techniques [36,37,38,39,40]. The WG core may be reduced to a submicron cross-section while still sustaining single-mode propagation at 1.3–1.5 μm telecommunications wavelengths thanks to the extremely high refractive index difference between the silicon core (*n* = 3.5) and silica cladding (1.45) [13]. The smallest bending radius can be brought down to the micron range owing to such intense light confinement, providing a path to the realization of very dense photonic integrated circuits on a single silicon chip. Due to the increased contact of the WG mode with the sidewall surface roughness, such high light confinement in submicron SOI strip WGs also produces noticeably increased propagation losses. Surface roughness is the cause of large propagation losses, which might make it impossible to design compact integrated circuits, according to extensive experimental research. High bending losses, usually in the range of 1 dB per 90-degree bend, are also caused by the same surface roughness [41].

**Figure 3:**
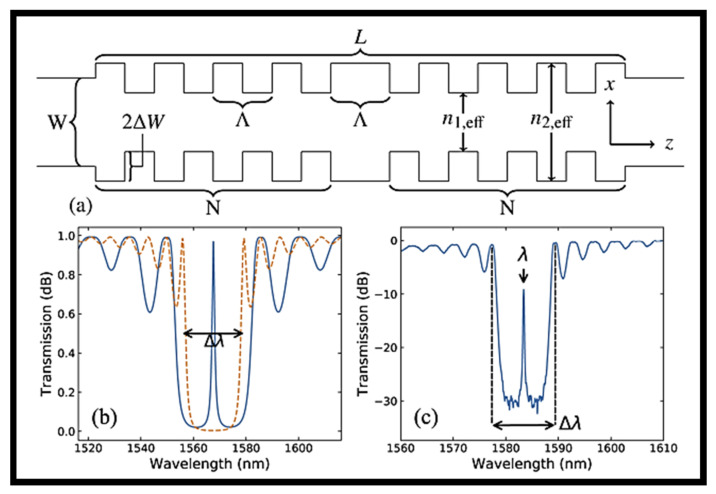
Phase shifted BG. (**a**) A quarter-wave phase-shifted BG with N = 50% duty cycle square wave variations of the period along both sides of a length phase shift facet. Adapted with permission from [43]. (**b**) Transmission of a SiN BG using the TMM model with (solid) and without (dashed) quarter-wave phase shift. Adapted with permission from [43]. (**c**) The response of a phase-shifted BG with the distinctive transmission peak at the center wavelength. Adapted with permission from [43].

Designing single-mode distributed Bragg reflector (DBR) and distributed feedback (DFB) lasers, for instance, requires the capacity to spatially modulate gratings and apodize their response [31]. It has been demonstrated that a λ/4 phase shift in DFB lasers increases the mode stability at the center wavelength [42]. On the AIM Photonics 300 mm Si photonics foundry line, phase-shifted SiN BGs were produced utilizing 193 nm deep ultraviolet lithography (DUVL) [43]. A BG structure with square corrugations on both sides is shown in Figure 3a,b, showing an analogous TMM model of gratings with (solid) and without (dashed) the λ/4-phase-shift. A characteristic experimentally determined optical transmission spectrum of a phase-shifted BG is shown in Figure 3c [43].

The effective writing of a BG in a fiber core, in particular, has greatly expanded its technical uses. A traditional grating device, on the other hand, is normally intended for a specific use, limiting general-purpose applications since the index modulation profile is defined after manufacture. Field programming is used to construct a completely programmable grating that is both fast and electrically changeable [44]. The notion is shown by building an integrated grating on an SOI substrate that can perform several signal processing operations such as temporal differentiation, microwave time delay, and frequency identification. The advent of ultrafast and customizable gratings has opened up new possibilities for programmable optical data processing at light speed.

### 3.2. Recent Advances in Si-Based BG Structures

Flexible spectrum tailoring may be accomplished with BGs. It has been demonstrated that by modulating the coupling coefficient (grating strength) along the grating, a filter [45,46] with any spectral response can be built [47]. This approach, also known as apodization, has allowed for the creation of adaptable spectrum filters for applications such as WDM [48,49], optical signal processing [50], optical communications [51], and astrophotonics [52], to mention a few. Bragg filters have traditionally been realized in integrated photonic systems using sidewall corrugated gratings, which modulate the WG width. This approach is constrained by the manufacturing process’s minimum corrugation size, which dictates the smallest disruption that can be accomplished, as well as the minimum coupling coefficient and filter bandwidth (BW). Because of the strong RI contrast, this limitation is extremely crucial in the SOI platform, where the filter performance is very responsive to tiny fluctuations in the corrugation width. In a 220 nm-thick SOI platform, for example, sub-nanometer BWs need WG sidewall corrugations of 10 nm or less, which are difficult to produce [36]. New modulation approaches, such as misaligned sidewall corrugations, grating pitch modulation [53], and subwavelength-tailored sidewall gratings [12], have been developed to alleviate this restriction. These approaches have been used to demonstrate Photonic Hilbert transformers [54,55] and multi-channel filters [51,56]. Nevertheless, corrugation widths that are difficult to produce in a consistent manner (less than 20 nm) are usually needed [51,56].

Cladding-modulated BGs, or structures with periodic fluctuation physically segregated from the WG core, are an intriguing option for implementing spectrum filters in silicon WGs. On lateral Si strips parallel to the WG, periodic arrays of lateral cylinders [57] and sidewall corrugations [58] have been suggested. By carefully arranging the grating in close vicinity to the WG core, these geometries enable the creation of weak BGs with limited spectral characteristics. Concurrently, designs with more relaxed minimum feature sizes can be created. Based on cladding-modulated gratings, Gaussian-apodized BGs [59] and multi-band filters [60] have been realized. In Si WGs with laterally connected Bragg loading segments, a unique geometry for designing complicated Bragg filters with an adjustable spectrum response is suggested [61]. The WG core is built with a delocalized mode field, which minimizes the sensitivity to manufacturing faults and increases the quality of synthesized coupling coefficients and the spectral shape control. The authors offer an effective design technique for cladding-modulated BGs that uses layer-peeling and layer-adding algorithms to easily synthesize an arbitrary target spectrum. By building and experimentally proving a complex spectrum filter on an SOI platform with 20 non-uniformly spaced spectral notches with a 3 dB linewidth as narrow as 210 pm, the suggested filter idea and design approach are proven [61].

**Figure 4:**
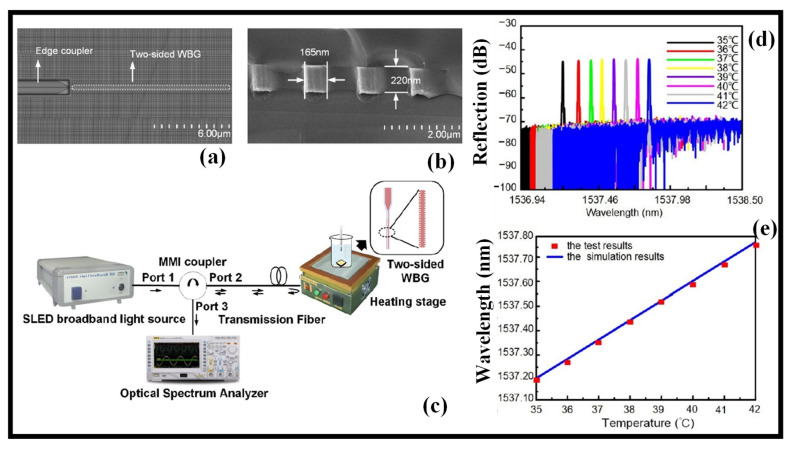
SOI WG-based BG. (**a**) SEM image of the BG WG [62], (**b**) cross-sectional view of the BG WG. Adapted with permission from [62], (**c**) experimental setup. Adapted with permission from [62], (**d**) BG WG output spectrum at different temperatures. Adapted with permission from [62], (**e**) wavelength-to-temperature plot. Adapted with permission from [62].

There is a demonstration of a high-sensitivity SOI WG-based-BG sensor [62]. The temperature measuring range of the BG sensor was wide, and the accuracy was great. It could monitor temperature changes in the human body by continually measuring temperature variations in the range of 35–42 °C. Biomedical sensing, forensic examination, microbiological research, drug screening, environmental monitoring, chemical synthesis, and other areas might all benefit from the sensor. Unlike FBG, which required photosensitive materials in the fiber to be exposed to UV light, WG BG only required etching periodic geometric shapes on the WG’s surface or side to produce a periodic effective refractive index (*n_eff_*) distribution of the grating, which has the benefit of a small volume and seamless integration. The SEM images of the BG structure top view and the cross-sectional view are shown in Figure 4a,b, respectively. The broadband light source was linked to port 1 of an MMI coupler by an optical fiber from a super radiant LED. To decrease coupling losses, Port 2 of the MMI coupler was connected to the BG WG laterally using tapered fiber optics. Light is selectively reflected by the BG WG. Through three ports of the MMI coupler, the light reflected from the BG WG was coupled to the spectrum analyzer. A spectrograph was used to measure the BG WG’s reflected wavelength and 3 dB BW. On the loading platform of the heating stage, the BG WG was placed. Figure 4c depicts a schematic diagram of the experimental setup. The heating stage was set at 35–42 degrees Celsius, with a 1-degree Celsius heating interval. As illustrated in Figure 4d, the spectrum reflected by the sensor performs a redshift as the ambient temperature increases [62]. With the mounting temperature, the BG WG’s central wavelength redshifted and grew linearly. A linear fitting was carried out. The device’s temperature sensitivity is 80 pm/°C, and the findings are displayed in Figure 4e [62].

## 4. BG Structures Based on a Polymer Platform

Polymers have a molecular structure that consists of extended chains of 1-D covalent bonds connected by secondary bonding networks. Polymer structural uniqueness provides special attributes such as structural flexibility, high-temperature dependency, and low thermal conductivity; nevertheless, it also entails low thermal stability and chemical instability such as photo-oxidation. The issue of the optical loss of polymers near optical communication windows owing to vibrational overtone absorption has been explored using fluorinated polymers [63]. The propagation loss of fluorinated polymers has been reduced below 0.1 dB/cm thanks to organic chemistry [64,65]. Due to the impact of a lower electron energy level, the stronger C–F bond enhances thermal and chemical stability over the C–H bond [66,67]. As a result, fluorinated polymers have substantially better thermal stability at temperatures above 350 °C, and no photo-oxidation has been reported in WDM optical communication devices running at a high optical power [68,69].

### 4.1. Polymer Materials and Fabrication Methods

New kinds of polymers for optical and photonics applications have been created in numerous laboratories throughout the world in recent decades, and some of them are already commercially accessible [70,71,72,73,74,75,76,77,78]. A few important polymer materials are listed in Table 1. Low optical losses at operating wavelengths (comprising the IR spectrum), well-controlled and tunable refractive indices, thermal and chemical resistance, mechanical and environmental stability, and environmentally friendly assembly methods are among the distinctive and outstanding optical properties of these new polymers [79,80,81]. Optical planar WGs are fundamental building blocks for the implementation of optics and photonic devices, and numerous manufacturing procedures for polymer WGs have been published. Mask photolithographic technology and a subsequent wet etching process, photoresist patterning (especially in combination with reactive ion etching (RIE)) [82], two-photon-polymerization [83], direct laser writing (DLW) [84], E-beam writing [85], flexographic and inkjet printing [86], a hot embossing process [87], photo-bleaching [88], and other fabrication methods are among them. Many processing stages are involved in these technologies, which might result in extended fabrication durations and low yields. As a result, technologies such as stamping processes are being researched for large manufacturing. Roll-to-roll (R2R) NIL [89] and roll-to-plate (R2P) NIL [90] are two of these technologies. These roller-based solutions allow for the use of polymers, which are the ideal material options for applications that need low-cost mass manufacturing. Flexible electronics are manufactured using these methods. The polymer platform does, however, have significant disadvantages, such as a low refractive index difference between the core and cladding. This restricts the confinement of light in a core and, as a result, the downsizing of integrated polymer devices. Additionally, active properties such as light emission, amplification, modulation, etc. are absent from polymers.

The production of optical WGs is illustrated by utilizing an R2P NIL, which is a kind of imprinting that uses a roller-mounded stamp (imprinting plate) and a rigid surface plate to attach the substrate [91]. The R2P manufacturing device is built around a transparent cylinder with a UV source in the center. The peeling-like separation process of the imprinting plate and substrate is a particular benefit of roller-based imprinting. This makes it easier to replicate complicated structures and imprint them on huge regions. The cut-back method was used to measure the WGs’ optical losses at wavelengths of 532, 650, 850, 1310, and 1550 nm. Optical losses at 850 nm were 0.19 dB/cm on average, whereas optical losses at 1310 nm were 0.42 dB/cm and 0.25 dB/cm at 650 nm, respectively. NIL has been shown to have considerable promise for the realization of polymer WGs, not just for optical connectivity applications.

**Figure 5:**
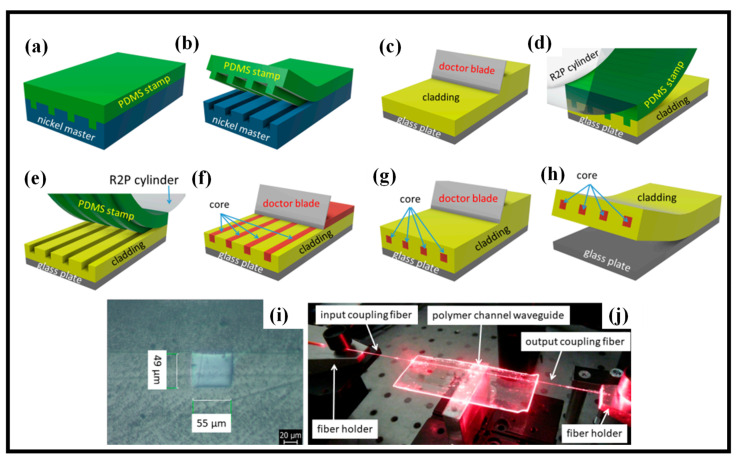
Manufacturing of the multimode optical WGs by utilizing R2P NIL. (**a**) Manufacturing of the PDMS stamp layer. Adapted with permission from [91], (**b**) separating stamp and Ni-mold. Adapted with permission from [91], (**c**) manufacturing of the Varnish 311 UV layer. Adapted with permission from [91], (**d**,**e**) manufacturing of the U-groove into the Varnish 311 UV substrate layer by the R2P process. Adapted with permission from [91], (**f**) manufacturing of the core layer into the U-groove Varnish 311 substrate. Adapted with permission from [91], (**g**) manufacturing of the Varnish 311 UV cover cladding layer. Adapted with permission from [91], (**h**) untying the WG structure from the glass substrate. Adapted with permission from [91], (**i**) optical microscope picture of the WG. Adapted with permission from [91], (**j**) light at 650 nm propagating in the WG. Adapted with permission from [91].

The following was the fabrication procedure [92]: The PDMS elastomer stamp was made with the help of a nickel master mold. The galvanoplastic procedure of a photoresist master created by the lithographic method was used to create this nickel-negative mold. The mold was 8 cm long, with 12 channels measuring 50 × 50 μm and a 250 μm pitch between them. The Sylgard 184 elastomer was used to make PDMS stamps, and the elastomer was made by combining the A and B agents in a 10:1 ratio, stirring the slurry, and then vacuuming it for 60 min in a centrifuge tube. The elastomer was then poured into the nickel mold and hardened in the oven for 20 min at 125 degrees Celsius, as shown in Figure 5a. The PDMS stamp was gently peeled away from the nickel mold after cooling (Figure 5b). The PDMS stamp was then mounted on the R2P machine’s cylinder. The polymer Lumogen OVD Varnish 311 cladding layer was then placed onto the glass substrate utilizing the doctor blade process, with a thickness of 500 μm. (Figure 5c). Before the imprinting process began, the R2P machine was correctly set up. The UV light intensity, the position of the cylinder height, and the imprinting speed seem to be the most critical characteristics. The R2P NIL tool’s UV light source is made up of 395 nm LEDs. The PDMS stamp was imprinted onto the Varnish 311 UV photopolymer once all the R2P machine’s settings were established (Figure 5d,e). The UV-curable inorganic-organic hybrid polymer was then applied into the U-grooves of the Varnish substrate by doctor blading, as shown in Figure 5f. UV radiation at 365 nm was used to harden the core layer for 60 s. Then, using the doctor blading approach, a Varnish 311 UV photopolymer cover cladding layer was manufactured, as shown in Figure 5g, and the WG structure was pulled away from the glass substrate (Figure 5h) [91]. The optical microscope picture of the WG is shown in Figure 5i. The broadcast of red light (650 nm) coupled with the optical fiber into the WG is seen in Figure 5j, which gives a full view of the optical losses measuring setup [91].

### 4.2. BGs Based on Polymer Materials as a Filter and Sensing Devices

Polymer materials-based optical devices offer several distinguishing characteristics, including a large thermo-optic (TO) effect, RI tunability by solution mixing, structural variety, freestanding flexibility, and adjustable material birefringence. These polymeric special properties have been used to demonstrate polymeric-integrated optic devices including novel TO devices based on large index tunability, single-mode WGs with substantially different core diameters, flexible functional WG devices, and birefringence-modulated polarization-controlling devices [92]. The fluorinated polymer exhibits high processibility for the manufacture of integrated optic WG devices made up of multilayers of thin-film polymers. ZPU polymers may be used to create polymer layers of varied thicknesses (1–50 µm), utilizing a spin coating and UV curing procedures that do not involve any major chemical treatment or heat sequence [93].

In recent years, a variety of devices built utilizing WG-based BGs employing various technical approaches have been exhibited. The direct E-beam writing procedure was used to create a tunable epoxy polymer SU-8 WG-based BG passband filter [94,95]. BG wavelength tuning by temperature variations was demonstrated by Liu et al. [96]. The polymer grating-assisted coupler filter boosts tuning efficiency even further and provides a 301 nm tuning capability [97]. WG-based BGs may also be used as add/drop multiplexers in WDM optical transmitters [98,99], fiber couplers [100], or mode converters [101]. BG operates in a single-mode range in the vast majority of documented applications [102]. Its usage in the few-mode and multimode regimes, which give a larger band, was also discussed. WG-based BGs are frequently utilized as optical sensors of temperature [103], strain [104], and humidity, in addition to their employment in optical telecommunication. WG-based BGs can be employed as sensing devices because of their typical properties, such as their big TO coefficient, increased Young’s modulus, or water absorption.

Tunable-wavelength lasers and wavelength-selective filters, which are essential parts of WDM optical communication systems, have been implemented using polymeric BGs WGs [98,105,106]. If wavelength-tunable lasers are used as an alternative to numerous fixed-wavelength light source modules for each color, different wavelengths may be selected using the same device, lowering system preservation and inventory expenses. Tunable-wavelength filters (TWFs) help select and extract the desired wavelength from a WDM signal; small BWs and broad tuning ranges are recommended for this application to maximize the number of communication channels [107].

Many strategies for forming WG-based BG structures in various types of polymers have been presented in recent times. Contact UV-photolithography is a typical lithographic process in which the grating constant periodicity in microns is determined by the diffraction limit of the light exposure. It is feasible to create tiny grating constants in hundreds of nanometers or fewer using modern X-ray photolithography (XRL) [108], electron beam lithography (EBL) [109], a focused ion beam (FIB), or nanoimprinting lithography (NIL) [110]. During the last few decades, DLW based on femtosecond (fs) pulse-induced light–matter interaction has grown significantly [111,112]. The ability to leverage numerous nonlinear light–matter interaction regimes as well as manage the thermal component of the process are fundamental benefits of employing fs lasers for DLW. The design, manufacture, and characterization of two types of multimode WG-based BGs for informatics and sensor applications are created by polymer PMMA-glass materials (hybrid composition) and the polymer SU-8 material (mono structure) using DLW [113].

**Figure 6:**
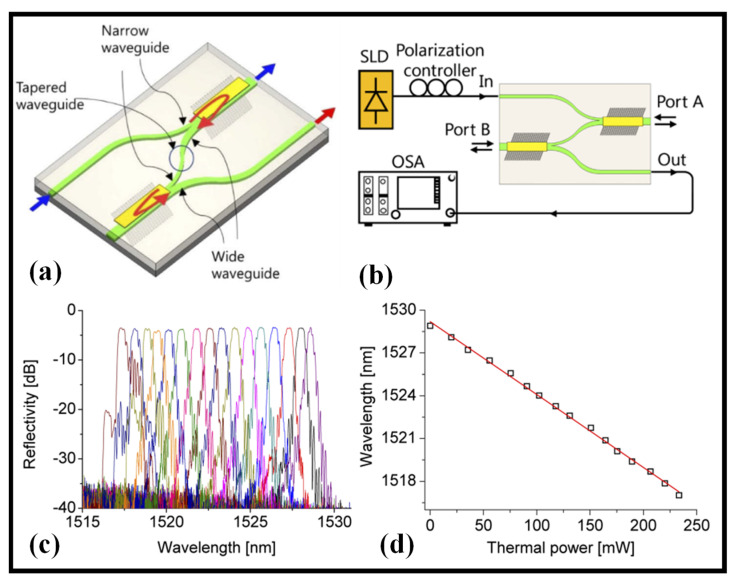
TWFs based on polymer WG. (**a**) Two-stage cascaded TWF. Adapted with permission from [107], (**b**) reflection spectrum measurement setup. Adapted with permission from [107], (**c**) reflection spectra taken by utilizing the heating power on the integrated microheaters in every step. Adapted with permission from [107], (**d**) ultimate wavelength of the reflection bands for the utilized thermal power. Adapted with permission from [107].

Without the need for an external circulator, the combination of a mode-sorting WG with a slanted BG allows for the extraction of Bragg reflected signals to another channel. Furthermore, the two-stage cascaded structure’s twofold reflection results in shorter reflection BW enhanced side mode suppression ratio (SMSR) characteristics and minimized adjacent-channel crosstalk by suppressing undesirable mode coupling. Over the whole wavelength tuning range, the device has a 20 dB BW of 1.0 nm and an SMSR of 35 dB [107]. Figure 6a depicts a cascaded tilted BG. A WG taper is used to transfer the reflected wave from the first phase into the second asymmetric Y-narrow branch’s WG. The odd mode input is then reflected onto an even mode output through the broad WG linked to the output port via the second slanted grating [107]. The measurement setup is displayed in Figure 6b [107]. The heating power on both electrodes was boosted by 15.8 mW in each step while preserving the original bias power, resulting in a wavelength setting of 0.8 nm for each step. The reflection bands were determined as displayed in Figure 6c [107]. As shown in Figure 6d, the peak wavelength of the reflection spectra was acquired concerning the applied power, and the tuning efficacy was 54 nm/W [107].

**Figure 7:**
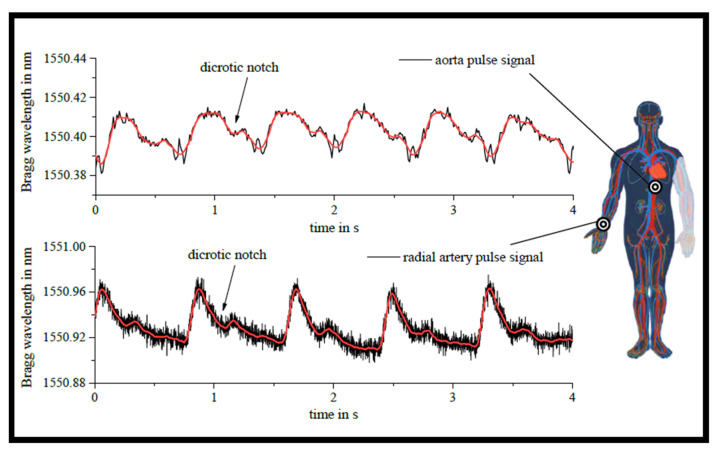
Epoxy-based sensor pad employed for examining the aorta and radial artery pulse signs of a patient. Adapted with permission from [114].

WG-based BGs made of flexible epoxy are constructed on a low-modulus TPX^TM^ polymethyl pentene polyolefin substrate for a simple-to-make and low-cost optomechanical sensor pad with a wide range of applications [114]. UV mask lithography is used to create rectangular EpoCore negative resist strip WGs. By totally changing the local refractive indices by a well-defined KrF excimer laser irradiation +1/−1 order phase mask, very persistent BGs are engraved straight into the channel WGs. An optical pickup for acoustic instruments, a broadband optical accelerometer, and a biomedical vital sign sensor measuring both respiration and pulse simultaneously highlight the repeatable and massively diverse sensing capabilities of an easy-to-apply optomechanical sensor pad. The TPX^TM^ substrate backside is provided with a double-sided surgical tape to permit full physical contact with the test person’s body area of concern in order to use the sensor pad for crucial sign examining in an easy-to-apply manner. In theory, numerous sensor placements near major arteries such as the aorta, radial artery, carotid, and femoral artery might produce a change in the *n_eff_* and grating period for medical pulse sign monitoring. Figure 7 shows the performance of the created optomechanical sensor in measuring the pulse of a participant [114]. For aorta pulse measurement, the sensor pad is initially located in the middle of the torso. The radial artery pulse signal is then captured as well for comparison. The dynamic interrogation system tracks the basic TE-mode reflection. From a medical standpoint, both observed pulse wave patterns match the predicted shape. Most significantly, in both pictures, all pulse signals include the distinctive dicrotic notch in the pulse wave from heart valve action, suggesting proper sensor operation. The depicted pulse readings include frequencies of 90 bpm (aorta) and 75 bpm (radial artery), which may be tracked temporally using continuous averaging.

**Figure 8:**
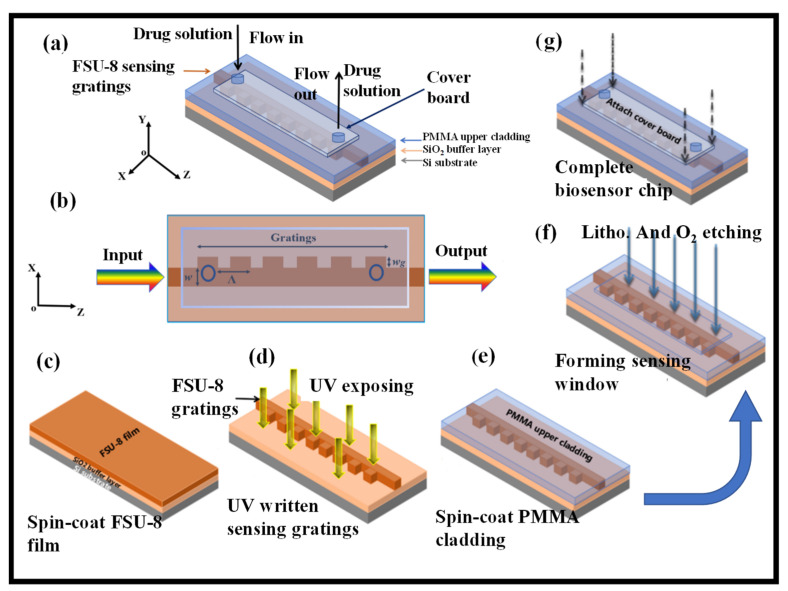
FSU-8 polymer BG biosensor. (**a**) 3D representation. Adapted with permission from [115], (**b**) top view. Fabrication process. Adapted with permission from [115]. (**c**) Spin-coated FSU film. Adapted with permission from [115], (**d**) UV written sensing gratings. Adapted with permission from [115], (**e**) spin-coat PMMA cladding. Adapted with permission from [115], (**f**) formation of the sensing window. Adapted with permission from [115], (**g**) biosensor chip. Adapted with permission from [115].

The direct UV writing approach was used to create a fluorinated co-polymer BG-based optical biosensor to determine effective solution concentrations of ginkgolide A for inhibiting PMVEC apoptosis [115]. The sensing polymer WG materials employed were low-loss FSU-8 and PMMA. The effectiveness of adhesion and capture between ginkgolide A and the polymer WG materials was improved thanks to the steady binding energy based on the hydrogen-bonding affinity. Pharmacological investigations were used to determine the effective medication concentration range (5–10 g/mL) of ginkgolide A for preventing PMVEC apoptosis. The grating-based biosensor’s true sensitivity was tested at 1606.2 nm/RIU. The detection limit was around 3 × 10^−5^ RIU, and the resolution was 0.05 nm [115]. The 3D representation and the top view of the polymer BG device are presented in Figure 8a,b, respectively.

Figure 8c–g depict the production process of the grating-based biosensor. The SiO_2_ buffer layer of the Si substrate was spin-coated with the FSU-8 negative photopolymer (Figure 8c). The FSU-8 thin film was then dried for 30 min at 95 degrees Celsius to eliminate surplus solvents before being scaled down to room temperature. The FSU-8 WG structure was then directly written using UV lithography equipment at 20 mW/cm^2^ for 6 s, using a contact exposure method to deposit photomask patterns onto the FSU-8 membrane. The sample was then post-baked at 100 °C for 1 h to excite photoinitiators in FSU-8 and create enough H+ to induce epoxy group crosslinking. A developer (PGMEA) solution was used to eliminate the uncrosslinked portion of the FSU-8 film. The optimal development time for the FSU-8 film thickness is 15 s. The etching rate is roughly 0.133 m/s on average (Figure 8d). The PMMA copolymer was then spin-coated, with the top cladding covering the WG gratings (Figure 8e). Reactive ion etching was used to remove the PMMA top cladding layer on the surface of the FSU-8 grating (Figure 8f). By regulating the etching rate, the sensing window was properly produced on the top surface of the BG WG. The cover board was then glued to the sensing window to produce the sensor’s microfluidic channel (Figure 8g).

## 5. BG Structures Based on the Plasmonic Platform

In this section, we have reviewed the recent advances in plasmonic BG structures, which are employed in eye-catching applications [14,116]. Several plasmonic technologies are now commercially available, while others are in the process of moving from the lab to the marketplace.

### 5.1. Plasmonics Fundamentals and Its Applications

**Figure 9:**
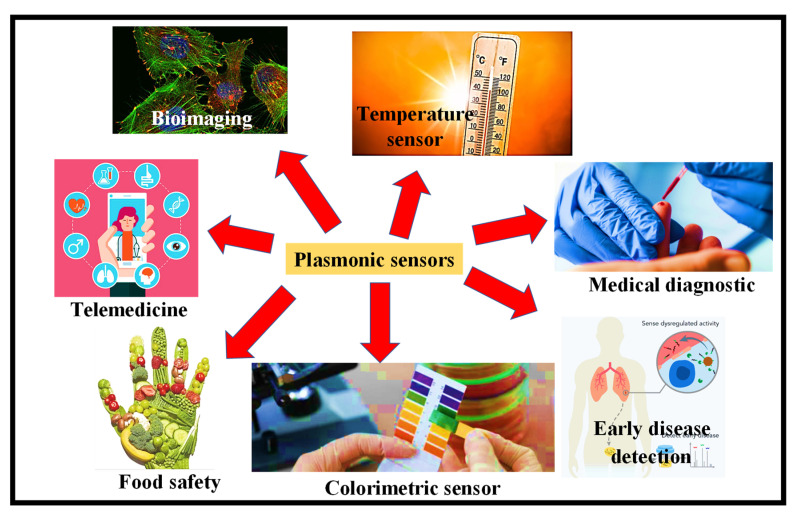
Utilizations of SPR sensors in bioimaging [133], temperature sensors [134], food safety [135], telemedicine [136], early disease detection [137], medical diagnostics [138], and colorimetric sensors [139].

The interaction between an electromagnetic (EM) field and the free electrons in a metal (usually gold or silver) that allow for the metal’s conductivity and optical characteristics is at the heart of all these technologies. When light strikes a metal’s surface, free electrons on the surface vibrate collectively, generating surface plasmons (SPs) [117]. The free electrons in a big piece of metal reflect the light that strikes them, giving the substance its luster. When a metal is only a few nanometers thick, however, its free electrons are constrained in a relatively limited region, which limits the frequency at which they may vibrate. The frequency of the oscillation is determined by the metal nanoparticle’s size. The plasmon absorbs only the percentage of incoming light (reflecting the rest of the light) that oscillates at the same frequency as the plasmon itself, a process known as resonance. This surface plasmon resonance (SPR) may be used to make nanoantennas, more efficient solar cells, and other useful products [118]. Food safety [119,120], colorimetric sensors [121], alcohol sensing [122], fuel adulteration [123], early ailment diagnosis [124], biosensing [125], medical diagnostics [126], pregnancy detection [127], bioimaging [128], telemedicine [129], underwater [130], a temperature sensor [131], and early disease detection are just a few of the applications for SPR sensors. Figure 9 depicts several of the most important uses for SPR sensors. Due to advancements in SPR technology, it has also been employed in electronic applications such as SPR imaging, optical filters, and modulators. In Japan, an SPP-based research report for the fast recognition of COVID-19 was recently released. Antibiotic-coated Au nanoparticles experience a resonance peak shift when the viruses are trapped, resulting in a unique color change [132]. Alternative methods are routinely used in pregnancy testing.

Sensors for detecting chemical and biological substances are one of the most well-studied uses of plasmonic materials [140,141]. One method involves coating a plasmonic nanomaterial with a chemical that binds to a target molecule, such as a bacterial toxin. Light shining on the material is reemitted at a certain angle in the absence of the toxin. However, if the toxin is there, it will change the frequency of the surface plasmon and, as a result, the angle at which light is reflected. This impact may be quantified with remarkable precision, allowing for the detection and measurement of even negligible amounts of toxin [141]. Several start-ups are working on products based on this and related ideas, including an internal sensor for batteries that can track their activity and help increase the power density and charge rate, as well as a device that can detect viral bacterial illnesses. Plasmonics is also making inroads into disk-based magnetic memory storage. Heat-assisted magnetic recording technologies, for example, boost memory storage by heating small regions on a disk for a brief period while writing [142].

As enterprises seek to capitalize on plasmonics, they must guarantee that their products are fairly priced, dependable, durable, and easy to scale up and combine with other components. Despite these obstacles, the future appears promising. Plasmonics experts may now employ materials other than gold (Au) and silver (Ag), such as graphene and semiconductors, thanks to the development of metamaterials, which are synthetic nanoscale materials in which plasmons cause strange visual phenomena [143]. According to a recent market analysis conducted by Future Market Insights (FMI), the worldwide SPR market is anticipated to be worth USD 910.4 million in 2022 and USD 1.5 billion by 2029 [144].

### 5.2. Types of Plasmonic WGs

In recent times, plasmonic WGs such as insulator–metal–insulator (IMI), metal–insulator–metal (MIM) [145,146], metal grooves [147], metal strips [148], metal wedges [149], and hybrid plasmonic WGs [150] have been proposed. These WGs restrict EM-waves close to the surface, beyond the light’s diffraction limit. It is commonly known that the IMI WG has less of a propagation loss than the MIM WG, resulting in a longer propagation length [151]. The MIM WG, conversely, is superior to the IMI WG in terms of confining light waves. Likewise, as wavelength-dependent photonic devices, BGs based on the IMI WG have been the subject of several studies [152,153]. However, because of their desirable property of light confinement further than the diffraction limit, MIM WGs have potential for nanoscale applications. MIM has a higher transmission loss, although it may be ignored in nanoscale devices [154]. Gordon [155] and Dionne et al. published thorough analytical analyses on MIM WGs in 2006 [153], while Bozhevolnyi et al. showed channel plasmon subwavelength WG elements such as interferometers and ring resonators [156]. Several innovative photonic devices based on SPPs have been studied in recent years, including triangular WGs [157], power splitters [158], plasmonic sensors [159,160,161,162], absorption switches [163], Bragg reflectors [164], plasmonic color filters [165], and absorbers [166].

### 5.3. Plasmonic Materials

Metals are the most widely utilized materials for plasmonics in near-infrared (NIR) to the long-wavelength visible range. Because of the good bulk dielectric characteristics of Au and Ag, the research on these metals was first centered on them. Noble metals can be used as ideal conductors when the frequency is lower than the NIR region [167,168]. EM waves cannot propagate because light may be perfectly reflected. In the visible and NIR ranges, the EM-wave penetrates the metal, increasing the loss. In this frequency range, SPPs can be produced [169]. Metals are transparent in the ultraviolet (UV) region, but high-energy photons can create photoelectrons, causing loss. Because of the electron bandgap transition, Au and Ag absorb a lot of light [170]. Even though Ag has a superior dispersion curve for plasmonics in the visible region, it requires protective coatings to avoid chemical and mechanical corrosion, limiting its use in biosensing. Au has become more popular for plasmonic sensors due to its chemical and mechanical stability. Surface plasmonic enhanced infrared vibrational spectroscopy and Raman scattering can be employed for biosensing in this spectral region [171,172].

Plasmonics employing new metals have gotten increased attention because of the enticing possibility of integrating plasmonic activity with attractive inherent targeted material features. The study of UV range plasmonics is now being pursued. Aluminum (Al) is a fascinating material from both a fundamental and an applied standpoint. In comparison to noble metals, it is a plentiful and inexpensive commodity. Al is explored more than the other probable UV plasmonic materials due to its economical prices [173,174]. In Al, a rather strong interband transition is restricted to a small energy range of approximately 1.5 eV. Al is quite Drude-like below and above this energy. More investigations on Al-based plasmonics are focused on UV-operated optical devices, such as optical and plasmonic integrated circuits [175] and color filters [176,177]. Surface plasmon-enhanced Raman scattering, absorption, and fluorescence can be employed for biosensing in this frequency range. Al is employed not only in UV plasmonics but also in the long-wavelength section of visible regime plasmonics for absorbance augmentation [178,179]. Since relevant design and manufacturing methods have matured, the use of plasmonics based on Al metal in biosensing is projected to skyrocket [173]. Plasmonic biosensors based on graphene that function in the infrared range have lately become a hot subject [180,181,182]. They have been utilized for vibrational spectroscopy [183] and as a gas sensor [184]. Other metal materials have also been combined with graphene [185,186]. The sensitivity of phase interrogation for single-stranded DNA was enhanced to 10^−18^ M by putting graphene on top of a thin coating of Au [187].

### 5.4. Plasmonic BG Filters and Sensors

A plasmonic BG is a one-dimensional optical device that may reflect a specific wavelength of light while transmitting all others due to periodic variations in the RI of the WG. The spectral response of a BG is regulated by setting the index modulation profile [14]. It can be employed as a flexible plasmonic filter and has experienced significant use in different scientific and industrial domains due to its easy design and unique filtering capacity. In this section, the filtering and sensing capabilities of plasmonic BGs are discussed by focusing on the research works published in recent years.

By incorporating the periodic thickness modulation of thin metal stripes implanted in a dielectric, Boltasseva et al. presented miniature and effective BGs for long-range SPP working at an operational wavelength of 1550 nm [188]. Many attempts have also been made to investigate BGs on MIM WGs [14,189,190,191]. On flat metallic surfaces, Wang and Wang introduced metal heterostructure SPP Bragg reflectors with nanocavities [189]. Hosseini and Massoud proposed a low-loss plasmonic Bragg reflector made up of alternately layered MIM WGs made of various dielectric materials [190]. Surface plasmon BGs were created by Han et al., utilizing a periodic fluctuation in the width of the dielectric in an MIM WG [192]. Hosseini and Massoud have also explored the distinctions between the index modulation and thickness modulation of MIM WG BGs. The grating periods of these structures range from 400 to 600 nanometers [191].

**Figure 10:**
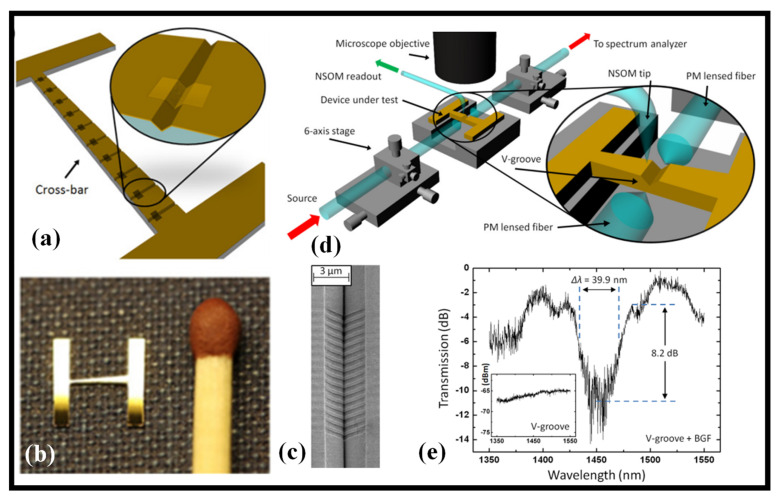
Plasmonic BG filter. (**a**) Graphical illustration of V-grooves along the device cross-bar. Adapted with permission from [193], (**b**) a 6 mm × 6 mm Au device adjacent to a matchstick. Adapted with permission from [193], (**c**) SEM photo of a V-groove comprising a BG filter. Adapted with permission from [193], (**d**) experimental system for NSOM and transmission measurements. Adapted with permission from [193], (**e**) transmission spectrum. Adapted with permission from [193].

In plasmonic V-groove WGs manufactured by wafer-scale processing based on NIL, spectrum filtering using state-of-the-art BGs is demonstrated [193]. Near telecommunications wavelengths, the transmission spectra of devices with 16 grating periods show the spectrum rejection of channel plasmon polaritons with an extinction ratio (ER) of 8.2 dB and a 3 dB BW of 39.9 nm. The oscillations of propagating modes through grating-less V-grooves match well with the n_eff_ values determined by FEM. The findings mark a step forward in the development of plasmonic V-grooves with increased functional diversity and mass-production viability. The grooves run the length of the crossbar in the H-like design of the devices, with the crossbar size set to allow for a range of groove lengths from 105 to 371 μm, as shown in Figure 10a [193]. A prototype of the device is illustrated in Figure 10b next to a standard matchstick [193]. Figure 10c shows an SEM picture of a V-groove BG filter [193]. Near-field scanning optical microscopy (NSOM) and transmission measurements are used to describe the devices using comparable but distinct experimental setups; a schematic is shown in Figure 10d. In both situations, two polarization-maintaining lensed optical fibers are employed for light launch and collecting in an end-fire arrangement with a TE-polarized light [193]. Figure 10e depicts transmission via a 311 μm-long V-groove BG filter which has a 3 dB BW of 39.9 nm and an 8.2 dB ER determined from the first reflection minima [193].

**Figure 11:**
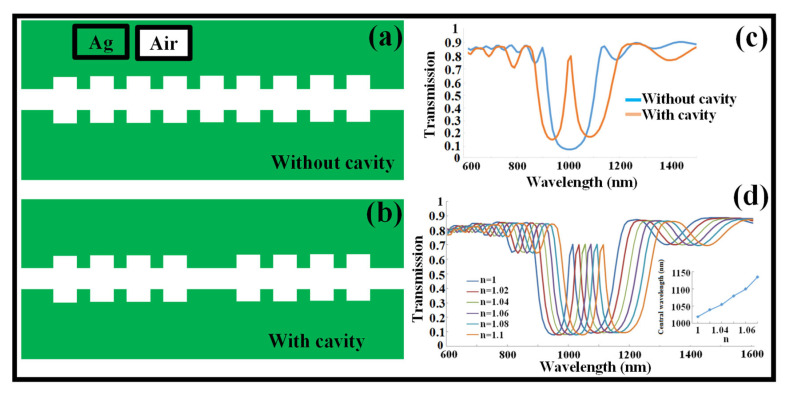
Plasmonic BG structure based on MIM WG. (**a**) BG without a cavity. Adapted with permission from [197], (**b**) BG with a cavity in the middle. Adapted with permission from [197], (**c**) transmission spectrum of a BG with and without a cavity. Adapted with permission from [197], (**d**) transmission spectrum of a BG with a cavity concerning the change in the ambient RI. Adapted with permission from [197].

Standard BG designs based on MIM WGs are used in a variety of telecommunications applications; however, knowing the performance of MIM WGs in the visible spectral region is also important. The period of the unit cell must be lowered to a range of 100–200 nm to achieve fundamental Bragg reflection in the visible range. Additionally, because the *n_eff_* increases as the operating wavelength decreases, a periodic structure with a significantly shorter period, such as less than 100 nm, is required. The dielectric grating on the metal substrate can be shaped using electron beam lithography (EBL) or focused ion beam lithography (FIBL), both of which result in a roundish edge effect [194,195]. The BG based on MIM WG functioning in the visible spectral range, which needs a very precise manufacturing process with a Λ of ~100 nm to 200 nm for the fundamental Bragg reflection, may be realized by employing a higher-order plasmonic Bragg reflection with a Λ of ~400 nm to 600 nm. It is demonstrated that these structures may also be used to produce a narrow reflection BW [196]. The finite element method (FEM) is used to construct and numerically simulate a nanoscale Bragg grating reflector created on the defect MIM WG, as shown in Figure 11a,b [197]. In transmission spectra, the MIM-based device provides a highly adjustable wide stopband. By adding a cavity in the middle of the DBR, the narrow transmission wavelength appears in the stopband, as shown in Figure 11c. By adjusting the geographical characteristics, the center wavelengths may be readily changed. With the advancement of SPP technology in metallic WG structures, additional options for manipulating light at deep sub-wavelengths are becoming available. As the RI of the ambient medium changes, a shift in the narrow transmission wavelength is observed in the transmission spectrum, as shown in Figure 11d. Hence, this device can be employed as an RI sensor [197].

**Figure 12:**
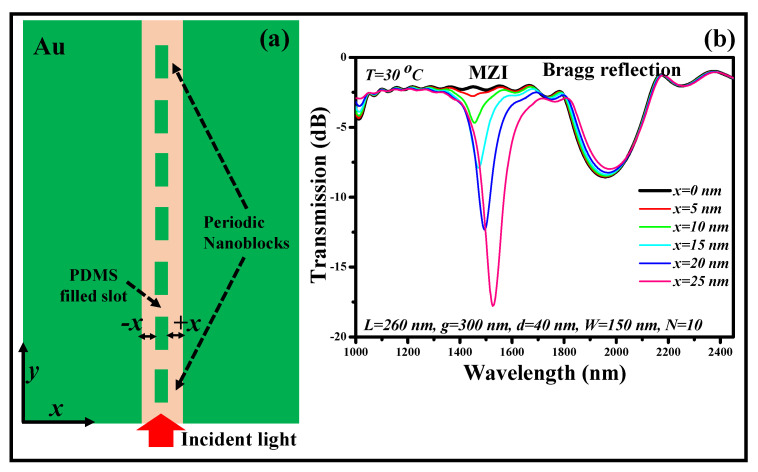
Modified BG structure established on an MIM WG. (**a**) Schematic representation. Adapted with permission from [198] and (**b**) transmission continuum of the modified BG formation. Adapted with permission from [198].

In our recent research work, we reported a numerical analysis of a modified BG structure realized on an MIM WG that may be used as an optical filter and a temperature sensor at the same time [198], as shown in Figure 12a. The conventional BG structure is made up of a regular pattern of nanoblocks (NBs) that are adorned in the center of the MIM WG, ensuring a large Bragg reflection BW. The NBs are relocated to one side of the MIM WG in the proposed research, generating a distinct optical path with uneven indices on both sides of the grating, resulting in a short MZI dip and the Bragg reflection spectrum, as shown in Figure 12b. The MIM WG is loaded with a temperature-sensitive polydimethylsiloxane (PDMS) polymer to functionalize the MZI dip for temperature-sensing applications. As a result, the modified BG structure may be used as a bandstop filter with a BW of 200 nm and a temperature sensitivity of 0.47 nm/°C [198].

A long-range surface plasmon polariton (LRSPP) WG-based thermally adjustable BG filter is presented and theoretically examined [199]. The Au stripe serves as an optical WG and a thermal heater at the same time. Along the path of light propagation, the width and height of the metal grating teeth alter at regular intervals. The light reflection and heat driving behavior are studied using eigenmode expansion (EME) and the finite element method (FEM), respectively. A reflectivity of 0.578 and a 3 dB BW of 1.1 nm may be reached thanks to the 3D arrangement. The peak wavelength in the reflection spectrum moves from 1549.9 nm to 1544.3 nm when the temperature rises from 25 to 75 degrees Celsius [199].

## 6. Pros and Cons of the Optical Platforms under Discussion

In this section, the advantages and disadvantages of the Si, polymer and plasmonic platforms are discussed. All three platforms are highly attractive; however, the advantages of using one can be greater than the others. This discussion will give a general idea to the researchers to make a wise decision while choosing an optical platform for the realization of optical devices.

Si platform—Given the potential advantages of optoelectronics integration, “Si photonics” has garnered major research efforts for more than ten years. Si photonics enables the more tightly integrated monolithic integration of several optical functionalities into a single device by using silicon as an optical medium and CMOS fabrication processing technologies. A single device can incorporate optical waveguides, modulators, and photodetectors, resulting in a reduced form factor. Light coupling capabilities at the input and output of a Si photonics IC are crucial to the success of the product because many current devices still need external laser sources. To make Si photonics devices commercially feasible and the go-to technology for future optoelectronic communication, designers still have a lot of obstacles to overcome. Moreover, the device fabrication cost is high due to the need for a cleanroom facility and sophisticated lithography and plasma etching equipment.

Polymer platform—The next generation of optoelectronic integrated circuits (OEICs) can be realized using polymer-based optical waveguide devices. These hybrid devices may be incorporated onto any current electronic substrate. A variety of monomers are combined in several different ways to create optical polymers. Many passive and active devices, as well as several promising polymer materials with appropriate optical and mechanical characteristics, have been developed. The use of polymers to create OEICs has several benefits, including cost-effective production based on tried-and-true techniques from the microelectronics industry. A polymer can also have a mix of photosensitivity, thermo-optical characteristics, and electro-optic nonlinearity. For the upcoming generation of optoelectronics systems, these characteristics enable the formation of new, multifunctional OEICs. However, polymers are unable to endure extremely high temperatures because, in contrast to metals, all polymers melt down very quickly. Moreover, polymers cannot be utilized in heat applications because of their extremely low heat capacity.

Plasmonic platform—The metallic coating used on a prism base or fiber core might be made of silver (Ag), gold (Au), or copper (Cu). Since Au has a larger real part of the dielectric constant than Ag, it shifts the resonance parameter more when the refractive index of the sensing layer changes. The development of islands in thin Au layers, band-to-band variations, and surface roughness brought about by thermal evaporation are some issues that are associated with Au, however [200]. Because of the oxidation, Ag’s chemical strength is weak, making it unable to achieve a repeatable result. As a result, the sensor continues to be erroneous. It is essential to coat the Ag surface in a thin, thick layer and cure it. Cu is a distinct plasmonic material with a nearly equivalent interband transition and optical dampening to Au in the 600–750 nm wavelength range. Tragically, Cu is also susceptible to oxidation. Recently, graphene has been used on top of Cu or Ag to prevent corrosion [201].

In the 40 years that plasmonic-based sensors have been developed, thousands of research articles and patents and tens of commercial products have been released. This is because these sensors outperform conventional ones in several ways, including (1) real-time monitoring to reveal the binding dynamics for observing various biological interactions between biomolecules, (2) label-free detection, (3) high reusability, (4) quick response times, (5) straightforward sample preparation, and (6) the use of few electrical components. 

Plasmonic sensors do, however, have some drawbacks, including (1) a nonspecific binding surface (which can be improved by immobilizing the analyte selective layer over the plasmonic film), (2) mass transportation restrictions, (3) steric hindrance during the binding event, and (4) the potential for data misinterpretation during routine events.

## 7. Outlook and Conclusions

Due to the fact that Si has already been used as the substrate for the majority of integrated circuits, Si photonic devices can be produced using commonly applied semiconductor fabrication methods. This makes it possible to produce hybrid devices that combine optical and electronic components on a single microchip. As a result, numerous electronics firms, including IBM and Intel, as well as university research organizations, are actively researching Si photonics to maintain Moore’s Law by employing optical interconnects to allow for faster data transfer both between and within microchips. Since the breakthrough of Bragg’s law in 1913, BGs have developed into vital optical components and are often used in a multitude of devices. The fast development of Si photonics, which is allowing for ground-breaking applications through tiny circuits produced using CMOS-compatible manufacturing techniques, is the driving force behind the interest in studying on-chip integrated gratings. As a result of the need to modify characteristics for each application, gratings built of Si and SiN have been demonstrated in a variety of forms, including corrugations and pillars on strip and ridge structures.

Optical planar WGs are fundamental building units for the implementation of optics and photonic systems, and numerous manufacturing procedures for polymer WGs have been published. Mask photolithographic technology and a subsequent wet etching method, photoresist patterning (especially in combination with RIE), two-photon-polymerization, DLW, E-beam writing, flexographic and inkjet printing, a hot embossing process, photo-bleaching, and other fabrication methods are among them. Many processing stages are involved in these technologies, which might result in extended fabrication durations and low yields. As a result, technologies such as stamping processes are being researched for large manufacturing. Roll-to-roll (R2R) NIL and roll-to-plate (R2P) NIL are two of these technologies. These roller-based solutions allow for the use of polymers, which are the ideal material options for applications that need low-cost mass manufacturing. Lately, there are several demonstrations of polymer WG BG devices that are employed in filtering and sensing applications. However, polymers are unable to endure extremely high temperatures because, in contrast to metals, all polymers melt down very quickly. Moreover, polymers cannot be utilized in heat applications because of their extremely low heat capacity.

Plasmonics make it possible to manipulate light in ways that go beyond the optical diffraction limit, which may be advantageous in applications including photonic devices, optical cloaking, biological sensing, filtering, and super-resolution imaging. The plasmonic platform is another attractive and viable solution for the formation of compact and highly sensitive devices, but plasmonic devices’ critical field-confinement potential is commonly accompanied by a parasitic Ohmic loss, which significantly lowers their effectiveness. Traditional BG designs based on MIM WGs are used in a variety of telecommunications applications; nevertheless, knowing the performance of MIM WGs in the visible spectral region is also vital. The period of the unit cell must be lowered to a range of 100–200 nm to achieve fundamental Bragg reflection in the visible range. Furthermore, because n_eff_ increases as the operating wavelength decreases, a periodic arrangement with a significantly smaller period, such as one less than 100 nm, is required. Therefore, it still requires some time to master the fabrication of plasmonic devices and light coupling methods, which makes it demanding due to its subwavelength dimensions. That is the reason why most of the plasmonic devices are numerically proven, yet their experimental verification is still a challenge.

The optical platforms studied in this review are highly attractive, and the photonic devices realized on them show good performance. However, according to the author’s opinion, there is still a need to research low-cost materials and fabrication procedures that can reduce the device cost in general [202,203,204,205]. For instance, the Si platform is well-explored, and the fabrication processes are well-optimized. However, it requires clean-room technology to manufacture the devices, which is unaffordable for low-budget laboratories or small-to-medium enterprises (SMEs). For that reason, coating techniques such as dip-coating, spin-coating, or spray-coating should be widely explored with the nano-imprint lithography (NIL) method to manufacture photonic devices in a single fabrication step in a standard laboratory setup.

## Figures and Tables

**Table 1:** Commercially available polymers for photonics applications. Adapted with permission from [70].

Polymer Type	Manufacturer	Optical Losses	Refractive Index
Cyclic olefin copolymer	Topas advanced polymers company	0.5 dB/cm at 830 nm, 0.7 dB/cm at 1550 nm	1.53
Cyclotone^TM^	DOW Chemical	0.81 dB/cm at 1300 nm	1.552 @ 633 nm, 1.537 at 1310 nm and 1.535 at 1550 nm
EpoCore	Micro resist technology GmbH	0.2 dB/cm at 850 nm	1.580 at 850 nm
Exguide^TM^	FOWG series from Chemoptics Inc. (Daejeon, Korea)	<0.1 dB/cm at 850 nm	1.547 at 830 nm
Truemode^TM^	Exxelis	0.04 dB/cm at 850 nm	1.57 at 633 nm
Ultradel 9120D	Amoco Chemicals	0.34 dB/cm at 850 nm, 0.43 dB/cm at 1300 nm	1.547 at 850 nm and 1.535 at 1550 nm
OE-4140 UV	Dow Corning	0.04 dB/cm at 850 nm	1.52 @ 850 nm

## Data Availability

Not applicable.

## Abbreviations

Bragg grating = BG; Waveguide = WG; Complementary metal oxide semiconductor = CMOS; Refractive index = RI; Bandwidth = BW; Extinction ratio = ER; Metal–Insulator–Metal = MIM; Insulator–Metal–Insulator = IMI; Reactive ion etching = RIE; Direct laser writing = DLW; Electron beam lithography = EBL; Ultraviolet = UV; Near-infrared = NIR; Roll-to-roll = R2R; Roll-to-plate = R2P; Nanoimprint lithography = NIL; Silicon = Si; Silicon nitride = SiN; Near-field scanning optical microscopy = NSOM; Polydimethylsiloxane = PDMS; Nanoblocks = NBs; Finite element method = FEM; Long-range surface plasmon polariton = LRSPP; Mach–Zehnder Interferometer = MZI; Focused ion beam lithography = FIBL; Eigenmode expansion (EME); Gold = Au; Silver = Ag; Aluminum = Al; Light-emitting diodes = LEDs; Fiber Bragg grating = FBG.
